# Efficacy of Sodium Channel-Selective Analgesics in Postoperative, Neuralgia, and Neuropathy-Related Pain Management: A Systematic Review and Literature Review

**DOI:** 10.3390/ijms26062460

**Published:** 2025-03-10

**Authors:** Athena Brooks, Anna Hornbach, Jade E. Smith, Noelle C. Garbaccio, Nathan Keller, Jessica Lemke, Jose A. Foppiani, Dominika Gavlasova, Theodore C. Lee, Marie-Claire Buckley, Umar Choudry, Samuel J. Lin

**Affiliations:** 1 Department of Surgery, Division of Plastic and Reconstructive Surgery, University of Minnesota, Minneapolis, MN 55455, USA; brooksa@umn.edu (A.B.); hornb078@umn.edu (A.H.); kell1879@umn.edu (N.K.); lemke298@umn.edu (J.L.); foppi002@umn.edu (J.A.F.); buckl012@umn.edu (M.-C.B.); choud008@umn.edu (U.C.); 2 Department of Surgery, Division of Plastic and Reconstructive Surgery, Beth Israel Deaconess Medical Center, Harvard Medical School, Boston, MA 02215, USAngarbacc@bidmc.harvard.edu (N.C.G.); 3 Institute of Clinical and Experimental Medicine, 140 21 Prague, Czech Republic; nika.gavlasova@seznam.cz; 4 Georgetown University, District of Columbia, Washington, DC 78626, USA; teddychinglee@gmail.com

**Keywords:** sodium channel-selective analgesics, pain management, innovation

## Abstract

Postoperative pain is a prevalent problem, often lasting from days to years. To minimize opioid use and associated risks of dependency, Enhanced Recovery After Surgery (ERAS) protocols increasingly incorporate multimodal analgesics. Sodium channel-selective blockers are a promising non-opioid alternative, yet their application in postoperative pain remains underexplored. This systematic review evaluates their efficacy in managing postoperative, neuropathic, and neuralgia-related pain. A systematic review was conducted using controlled keywords across multiple databases to identify studies on sodium channel-selective blockers published up to 2024. Eligible studies included clinical trials, observational studies, case series, and reports involving patients aged 18 or older. Data were extracted on therapeutic outcomes, dosages, complications, and comparisons with other analgesics. Five studies met the inclusion criteria, involving 804 patients, 81.58% of whom were women. One study addressed postoperative pain, while the remaining five focused on neuropathy- and neuralgia-related pain. All studies reported significant pain reduction in at least one treatment group compared with placebo. In the study on postoperative pain, the sodium channel-selective blocker significantly reduced pain scores without requiring opioid analgesia. Across all studies, only two patients needed concomitant opioid therapy, and one discontinued treatment due to adverse effects. Dosages varied, with no reports of severe complications. Comparative analyses showed that sodium channel-selective blockers were as effective, if not superior, to traditional pain medications in reducing pain intensity. Sodium channel-selective blockers demonstrate significant potential in pain management with minimal opioid reliance. While effective for neuropathic pain, further studies are essential to validate their role in acute postoperative settings and refine their use in multimodal analgesia regimens.

## 1. Introduction

Pain management after surgery is essential for recovery, both to optimize patient comfort and to prevent complications such as opioid dependence [1]. Approximately 6% of opioid-naïve patients remain on opioids 90 days after surgery, highlighting the risk of long-term opioid use even among those without prior exposure [2]. According to the National Institute on Drug Abuse (NIDA), 21% to 29% of patients prescribed opioids for chronic pain misuse them, and 8 to 12% develop opioid use disorder [3].

Designed to improve postoperative outcomes, Enhanced Recovery After Surgery (ERAS) protocols significantly vary across institutions, particularly with respect to opioid use. A 2022 systematic review of ERAS protocols for post-spinal surgery highlighted these variations: some protocols incorporated opioids in all pain management, whereas others reserved opioids for severe pain, opting instead for non-opioid analgesics and non-pharmaceutical strategies such as physical therapy and early ambulation [4]. Similar trends have been observed in ERAS protocols for post-cardiac surgery and post-craniotomy [5,6]. In the context of a nationwide opioid crisis, the lack of standardization for postoperative opioid use is concerning. Exploring effective options for pain management with non-opioid analgesics is essential to protect patient safety.

Various non-opioid medications have been explored for postoperative pain management, including NSAIDs, muscle relaxants, and local nerve blocks [7,8]. New to the pain management landscape, sodium channel-selective analgesics offer a promising approach. Sodium channel blockers take advantage of voltage-gated sodium channels in the pain signaling pathway; they specifically target the Na_V_1.7 and Na_V_1.8 channel subtypes, which are highly expressed in sensory neurons [9,10]. In animal models, Mueller et al. found Na_V_1.7 inhibitors combined with muscle relaxants significantly reduced pain hypersensitivity and pain-related behaviors in mice postoperatively [11]. Genetic studies have further demonstrated that patients lacking Na_V_1.7 channels are insensitive to pain [12,13].

Despite these promising laboratory findings, the application of sodium channel-selective analgesics in humans remains under-researched. Massachusetts General Hospital and the University of Texas Southwestern both published reviews of Enhanced Recovery after Surgery (ERAS) protocols in 2019 to examine postoperative pain management strategies but did not mention sodium channel-selective analgesics [14,15]. This omission may be due in part to the relatively recent development of these drugs, yet it highlights a missed opportunity to recognize their effectiveness in controlling postoperative pain.

Key questions remain regarding the efficacy of sodium channel-selective analgesics in humans, whether they can be used as standalone agents, and how they may be incorporated into multimodal analgesia regimens. The distinct roles of these agents in postoperative pain and chronic neuropathic pain are also important to distinguish, as these conditions follow different pain pathways. This systematic review aims to evaluate the current state of research on sodium channel-selective medication for pain management. By synthesizing available evidence, we aim to provide insight into their potential as a non-opioid alternative for pain control across diverse modalities

## 2. Methods

### 2.1. Literature Review

#### 2.1.1. Search Strategy

A thorough literature review was conducted utilizing advanced search techniques, including subject headings, controlled vocabulary, and keyword combinations, across PubMed/MEDLINE and Web of Science databases. The search spanned publications available up to January 2025.

#### 2.1.2. Study Selection

The review included studies that explored the relationship between molecular pathways and various pain conditions, particularly those involving chronic and neuropathic pain. Emphasis was placed on identifying key mechanisms, such as sodium channel activity, inflammatory mediators, and neural plasticity, that contribute to pain perception and persistence.

#### 2.1.3. Data Extraction/Synthesis

The findings were narratively synthesized to construct a preliminary framework for understanding the molecular mechanism of pain. This synthesis identified critical pathways, including the role of voltage-gated sodium channels (e.g., NaV1.7 and NaV1.8) in neuronal excitability, the impact of pro-inflammatory cytokines like IL-6 and TNF-α in sensitizing nociceptors, and the contribution of central and peripheral sensitization in chronic pain states.

### 2.2. Systematic Review

This study protocol was prospectively registered with PROSPERO. Completion of the study was performed in accordance with the Preferred Reporting Items for Systematic Reviews and Meta-Analysis (PRISMA) statement guidelines [16].

#### 2.2.1. Eligibility Criteria

The eligibility criteria for this systematic review included observational studies, clinical trials, and case series that examined the use of sodium-selective channel blockers in humans. Articles were required to discuss postoperative pain control or pain management in neuropathy and neuralgia. Studies were included if they were written in English, French, or Spanish and involved patients aged 18 years or older.

Articles were excluded if they were abstracts, editorials, commentaries, letters to the editor, or systematic reviews. Additionally, studies were excluded if they focused on animal models or did not include the use of sodium-selective channel blockers in humans.

#### 2.2.2. Search Strategy

A comprehensive literature review using subject headings, controlled vocabulary, and keywords was used to search MEDLINE (in Ovid), Embase, Web of Science, the Cochrane Central Register, and ClinicalTrials.gov.co for studies published until 2024. The search terms used were “sodium-selective analgesics” or “sodium-selective channel blockers” or “sodium-channel selective analgesics” and “post-operative pain management” or “non-opioid post-operative pain management”.

#### 2.2.3. Study Selection

The search results were uploaded to Covidence, a systematic review management software package used for literature screening [17]. A two-stage screening process was conducted for study selection. In the first step, two screeners with either a bachelor’s degree in science or medical degree independently reviewed the titles and abstracts. In the second step, the same two reviewers reviewed the full text and selected studies that fulfill the eligibility criteria. If discordances were present, then the two original reviewers along with two additional reviewers convened and agreed on the inclusion of a study.

#### 2.2.4. Data Extraction

The variables extracted from this systematic review were title, authors, country, journal, type of study, total number of patients, percentage of female patients, average age, body mass index, length of stay, sodium-selective channel blocker dosage, opioid or other pain control dosage, reduction in opioid consumption, pain score (0–10), complications, and type of procedure.

#### 2.2.5. Outcomes

The results of this systematic review focus on the outcomes following the use of sodium-blocker analgesics, specifically highlighting pain reduction, dosage, comparison with other pain medications, and adverse events.

#### 2.2.6. Quality Assessment

To assess each study’s internal validity, the National Institute of Health (NIH) quality assessment tool was utilized [18]. Each article was categorized as “Good”, “Fair”, or “Poor” based on methodologic and reporting criteria.

#### 2.2.7. Statistical Analysis

A qualitative synthesis was performed due to the heterogeneity of the topics covered in the studies in this systematic review.

## 3. Results

### 3.1. Literature Review

#### 3.1.1. Pain at the Molecular Level

Pain is a complex sensory and emotional experience that arises from the activation of specialized sensory neurons known as nociceptors. At the molecular level, pain perception is governed by a variety of ion channels, receptors, and intracellular signaling pathways that modulate neuronal excitability and neurotransmission. Among these molecular components, voltage-gated sodium (NaV) channels, transient receptor potential (TRP) channels, and key neurotransmitters such as glutamate, substance P, and calcitonin gene-related peptide (CGRP) play critical roles in the initiation and propagation of pain signals [19,20]. Pain pathways are divided into two primary types: nociceptive pain and neuropathic pain. Nociceptive pain arises from direct activation of peripheral nociceptors in response to tissue injury, while neuropathic pain results from dysfunction or damage to the nervous system itself. The molecular mechanisms underlying these pain types involve distinct but overlapping pathways, with sodium channels playing a pivotal role in both acute and chronic pain conditions [21].

#### 3.1.2. Voltage-Gated Sodium Channels in Pain Perception

Voltage-gated sodium channels (VGSCs) are critical transmembrane proteins that facilitate the propagation of action potentials in excitable cells, playing a crucial role in nociception and pain perception. Structurally, these channels are composed of a central pore-forming α-subunit, which determines ion selectivity and gating properties, and auxiliary β-subunits that modulate channel kinetics and expression. The α-subunit consists of four homologous domains (DI–DIV), each containing six transmembrane helices (S1–S6) [22]. The S4 helix acts as the voltage sensor, moving in response to changes in membrane potential, while the S5–S6 segments form the ion-selective pore. Upon depolarization, VGSCs undergo a conformational change that allows Na⁺ influx, triggering neuronal excitability and pain signaling [22].

Among the VGSC family, Na_V_1.7, Na_V_1.8, and Na_V_1.9 are predominantly expressed in nociceptive neurons of the dorsal root ganglion (DRG) and play distinct roles in pain transmission [22]. Na_V_1.7, encoded by SCN9A, is highly sensitive to small voltage fluctuations and amplifies subthreshold depolarizations, effectively acting as a gain-control mechanism for pain signals. Mutations in SCN9A can result in congenital insensitivity to pain or inherited erythromelalgia, a disorder characterized by severe burning pain. Studies indicate that Na_V_1.7-dependent nociceptive action potentials occur independently of endogenous opioid signaling, underscoring its role as a fundamental molecular driver of pain perception [22].

Na_V_1.8, encoded by SCN10A, is distinguished by its resistance to fast inactivation and preferential expression in small-diameter DRG neurons involved in chronic pain states. This channel is particularly implicated in inflammatory pain and is being targeted for novel analgesic strategies, such as VX-548, a selective Na_V_1.8 inhibitor designed to suppress hyperexcitability in acute pain pathways [23]. Additionally, melatonin has been found to attenuate chronic visceral pain by downregulating Na_V_1.8 expression, suggesting a neuromodulatory role in nociceptive processing [24].

Na_V_1.9, encoded by SCN11A, contributes to persistent sodium currents that influence resting membrane potential and excitability. Gain-of-function mutations in SCN11A lead to depolarization block and reduced pain sensitivity, whereas loss-of-function mutations are associated with increased pain syndromes. Recent optogenetic studies have further clarified the distinct contributions of Na_V_1.7, Na_V_1.8, and Na_V_1.9 to nocifensive behavior, providing insights into targeted analgesic interventions [25].

The molecular architecture of VGSCs has also been explored through chimeric protein expression in E. coli, facilitating functional studies on channel gating and drug interactions [26]. With increasing knowledge of VGSC structure–function relationships, the development of subtype-specific inhibitors represents a promising avenue for targeted pain medicine.

#### 3.1.3. TRP Channels in Nociception

Transient receptor potential (TRP) channels are critical components in the transduction of noxious stimuli, including thermal, mechanical, and chemical pain signals. Among them, TRPV1, TRPA1, and TRPM8 play pivotal roles in sensory neurons, particularly within dorsal root ganglia (DRG) and trigeminal ganglia [27,28,29,30,31]. TRPV1, often referred to as the capsaicin receptor, is activated by noxious heat above 43 °C, protons (low pH), and inflammatory mediators such as bradykinin and prostaglandins [27,28,29,30,31]. Upon activation, TRPV1 facilitates calcium influx, leading to neuronal depolarization and nociceptive signal propagation. Protein kinase A (PKA) and protein kinase C (PKC) phosphorylation enhance TRPV1 sensitivity, contributing to heat hyperalgesia under inflammatory conditions [27,28,29,30,31].

TRPA1, another critical TRP channel, is primarily activated by reactive oxygen species, environmental irritants such as allyl isothiocyanate (mustard oil), and noxious cold temperatures [27,28,29,30,31]. TRPA1 activation is closely linked to inflammatory pain, as cytokines including interleukin-1β (IL-1β) and tumor necrosis factor-alpha (TNF-α) enhance its expression in sensory neurons, leading to prolonged pain states. TRPM8, in contrast, is responsible for detecting innocuous and noxious cold stimuli, as well as cooling agents such as menthol, playing a role in both cold hypersensitivity and pain modulation [27,28,29,30,31]. While TRPM8 is largely considered an anti-nociceptive channel, its downregulation has been associated with neuropathic pain conditions.

The interplay between TRP channels and inflammatory mediators significantly amplifies pain perception. For example, IL-6 and TNF-α upregulate TRPV1 expression in sensory neurons, leading to sustained hyperalgesia [27,28,29,30,31]. Furthermore, PGE2 enhances TRPV1 phosphorylation via EP4 receptor activation, lowering its activation threshold and intensifying pain sensitivity. These molecular interactions highlight the complex regulatory mechanisms underlying nociceptive processing.

#### 3.1.4. Inflammatory Mediators and Sensitization

Cytokines, chemokines, and prostaglandins are key mediators in inflammatory pain, acting to lower the activation threshold of nociceptive ion channels and prolong pain hypersensitivity. Among these, IL-6, IL-1β, and TNF-α are particularly significant in the sensitization of nociceptive neurons [32]. IL-6, signaling through the gp130/JAK/STAT3 pathway, has been shown to increase the expression of NaV1.7 and NaV1.8 sodium channels, leading to enhanced excitability in sensory neurons [32,33]. Additionally, IL-6 promotes astrocyte activation in the spinal cord, contributing to the persistence of inflammatory pain states [32,33].

TNF-α is another crucial mediator, acting through the TNFR1 and TNFR2 receptors to amplify pain signaling [34]. TNF-α upregulates NaV1.8 expression in DRG neurons, which, in turn, prolongs nociceptive transmission and contributes to thermal and mechanical hyperalgesia [34]. Furthermore, TNF-α enhances synaptic transmission in the dorsal horn by increasing glutamate release, thereby exacerbating excitatory nociceptive signaling [34]. IL-1β, another key cytokine, activates the p38 MAPK and NF-κB pathways, leading to increased expression of pro-nociceptive receptors such as TRPV1 and TRPA1 [34]. Additionally, IL-1β upregulates cyclooxygenase-2 (COX-2), promoting prostaglandin synthesis and amplifying inflammatory pain [34].

Prostaglandins, particularly PGE2, play a direct role in nociceptor sensitization. PGE2 binds to EP1–EP4 receptors, activating intracellular signaling pathways that potentiate sodium and TRP channel activity [35]. EP2 and EP4 receptor activation leads to PKA-mediated phosphorylation of TRPV1, lowering its activation threshold and enhancing pain perception [35]. Additionally, EP1 receptor signaling has been shown to reduce GABAergic inhibitory tone, increasing excitatory neurotransmission and prolonging hyperalgesia [35].

#### 3.1.5. Synaptic Transmission and Neurotransmitters in Pain Signaling

At the synaptic level, pain perception is regulated by excitatory and inhibitory neurotransmitters. Glutamate, the primary excitatory neurotransmitter in nociceptive pathways, binds to NMDA and AMPA receptors, triggering calcium influx and synaptic potentiation [36,37]. NMDA receptor activation plays a central role in long-term potentiation (LTP) within the dorsal horn, a mechanism implicated in the transition from acute to chronic pain [36,37]. Substance P, another excitatory neurotransmitter, binds to neurokinin-1 (NK1) receptors, facilitating synaptic plasticity and enhancing nociceptive signaling [36,37].

In contrast, inhibitory neurotransmitters such as GABA and glycine function to dampen pain transmission by hyperpolarizing dorsal horn neurons. However, pro-inflammatory cytokines such as TNF-α and IL-1β have been shown to impair GABAergic inhibition by downregulating GABAA receptor expression, leading to disinhibition and persistent pain states [36,37].

#### 3.1.6. The Role of Endogenous Opioids in Pain Modulation

Endogenous opioids, including enkephalins, endorphins, and dynorphins, exert analgesic effects by binding to opioid receptors (μ, δ, κ) in the central nervous system. Activation of μ-opioid receptors (MORs) inhibits adenylyl cyclase, reducing cyclic AMP (cAMP) levels and subsequently suppressing voltage-gated sodium channel activity [38,39]. MOR activation also hyperpolarizes neurons via G-protein-coupled inwardly rectifying potassium (GIRK) channels, reducing nociceptive transmission [38,39].

Recent studies have highlighted the impact of cytokines on opioid receptor function. IL-1β and TNF-α downregulate MOR expression in dorsal horn neurons, contributing to opioid tolerance and diminished analgesic efficacy over time [38,39]. This interaction underscores the importance of targeting both cytokines and opioid signaling pathways to develop effective pain therapeutics.

### 3.2. Systematic Review

**Figure 1:**
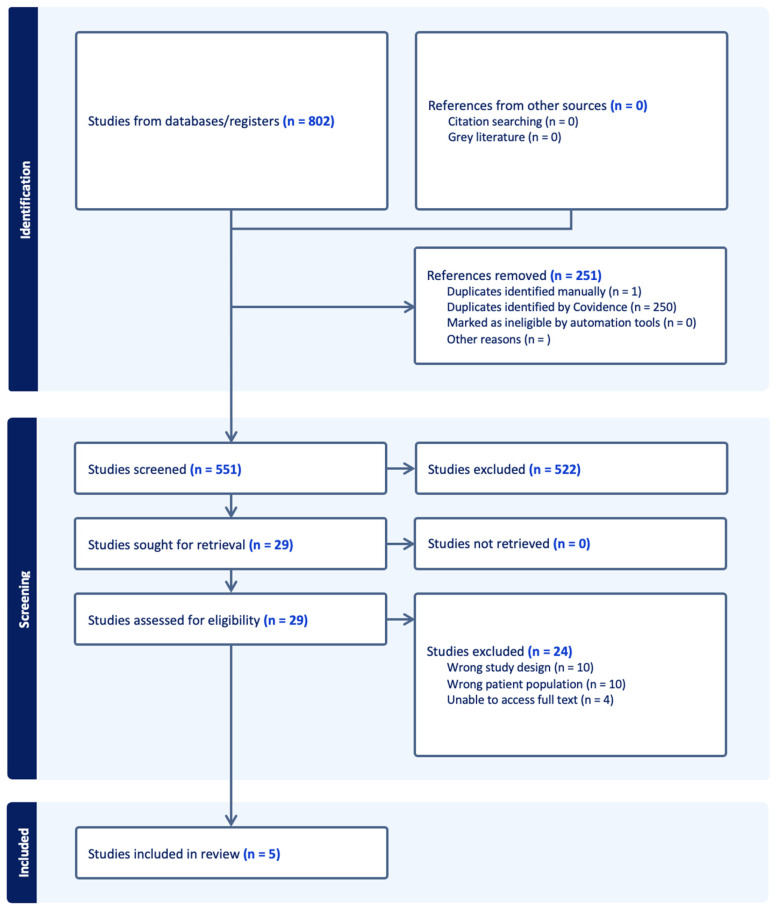
The PRISMA flow diagram.

The search yielded 551 titles, which were narrowed down to 29 articles following title and abstract screening. After a comprehensive full-text review, five studies met the inclusion criteria [40,41,42,43,44]. The PRISMA flow diagram is shown in Figure 1.

#### 3.2.1. Study Characteristics

This systematic review included five studies comprising 804 patients, 81.58% of whom were women. Four studies focused on neuropathy or neuralgia-related pain, including small fiber neuropathy (SFN), trigeminal neuralgia, and complex regional pain syndrome (CRPS), while one study investigated acute postoperative pain following abdominoplasty and bunionectomy. Sodium-selective channel blockers included VX-548, lacosamide, vixotrigine, ambroxol, and BIIB074. Study characteristics are shown in Table 1.

#### 3.2.2. Postoperative Pain Management

Jones et al. examined the efficacy of VX-548, an oral Na_V_1.8 channel inhibitor, for postoperative pain in patients undergoing abdominoplasty or bunionectomy [42]. Participants were randomized to receive high-dose VX-548, middle-dose VX-548, low-dose VX-548, oral hydrocodone-acetaminophen, or placebo. The highest dose of VX-548 significantly reduced pain over 48 h compared with placebo and opioid controls, with fewer participants discontinuing VX-548 due to lack of efficacy. Adverse effects, such as headache and constipation, were mild and more frequent in the post-abdominoplasty cohort.

#### 3.2.3. Neuropathic or Neuralgia-Related Pain

##### Small Fiber Neuropathy (SFN)

De Greef et al. conducted the LENSS study, a randomized, placebo-controlled trial evaluating lacosamide in SFN [40]. Lacosamide was titrated up to 200 mg BID over three weeks, and efficacy was measured using the Pain Intensity Numerical Rating Scale (PI-NRS). Significant reductions in pain intensity—reduction of at least 1 point—were observed in 58.3% of lacosamide-treated patients compared with 21.7% in the placebo group. Reductions in sleep interference and surface pain intensity were also observed. However, there were no significant changes in quality of life or autonomic symptoms, and increasing the dose to 300 mg BID for non-responders resulted in a higher incidence of adverse effects, including dizziness and gastrointestinal symptoms.

The CONVEY study, led by Faber et al., evaluated vixotrigine in idiopathic or diabetes-related SFN, focusing on its efficacy across different dosing regimens [41]. Of 265 patients in the open-label phase, 123 who achieved ≥30% pain reduction advanced to the double-blind phase, where participants were randomized to receive 200 mg BID, 350 mg BID, or placebo for 12 weeks. The 200 mg BID group showed significant reductions in average daily pain (ADP) scores compared with placebo, particularly in diabetes-related SFN, while the 350 mg BID group did not. More patients in the 350 mg BID group reported improved outcomes on the Patient Global Impression of Change (PGIC) scale compared with placebo (48.8% vs. 30.0%), though this difference was not statistically significant. Adverse effects, including falls, nasopharyngitis, and muscle spasms, were mild to moderate.

##### Complex Regional Pain Syndrome (CRPS)

Maihöfner et al. conducted a case series of eight patients evaluating the efficacy of topical ambroxol, a Na_V_1.8 channel blocker, in complex regional pain syndrome (CRPS) [43]. Similar to the study by de Greef et al., the Pain Intensity Numerical Rating Scale (PI-NRS) was used to assess treatment outcomes. All but one patient reported pain relief both at rest and during activity, with average reductions of 3 points at rest and 3.5 points during activity. The study noted variability in application frequency, and two patients used adjunct therapies, such as pregabalin or topical lidocaine, making it challenging to fully isolate ambroxol’s effects. As all patients had CRPS symptoms for less than 12 months, the efficacy of ambroxol for chronic CRPS remains unclear. Nonetheless, topical ambroxol may offer a promising, minimally invasive option for localized pain relief in early-stage CRPS.

##### Trigeminal Neuralgia

Zakrzewska et al. investigated BIIB074, a Na_V_1.7 selective blocker, in patients with trigeminal neuralgia [44]. The study was a phase 2a double-blind, placebo-controlled trial with 29 patients. Participants were randomized to receive 150 mg BIIB074 TID or placebo for 28 days. The BIIB074 group demonstrated significant reductions in paroxysms (45% placebo-adjusted reduction) and average daily pain (50% placebo-adjusted reduction). Clinician Global Impression of Change (CGIC) scores indicated improvement in 80% of BIIB074-treated patients compared with 36% in the placebo group. Adverse events, such as headache, nasopharyngitis, and sleep disturbances, were mild, with no severe or serious events reported. Study outcomes for all studies, including efficacy and safety findings, are detailed in Table 2.

#### 3.2.4. Quality Assessment

Three studies were rated as “good” based on methodological rigor and comprehensive reporting—the LENSS and CONVEY studies employed double-blinding and placebo-controlled designs, minimizing performance and detection biases, while the VX-548 study also included an active comparator and multiple dosing regimens, allowing for detailed comparisons of efficacy and tolerability. The Maihöfner et al. case series and the Zakrzewska et al. trial were rated as “fair”, primarily due to small sample sizes and, in the case of Maihöfner et al., the use of adjunct therapies, which complicated the isolation of ambroxol’s effects. Across studies, adverse events were comprehensively reported, and data on efficacy were collected using validated tools such as the Pain Intensity Numerical Rating Scale (PI-NRS) and Clinician Global Impression of Change (CGIC).

## 4. Discussion

Collectively, the five studies reviewed demonstrate the potential of sodium-selective channel blockers as effective, non-opioid options for pain management across various pain modalities, including acute postoperative pain and chronic neuralgia. Each study reported statistically significant pain reductions in at least one treatment group compared with placebo, underscoring their therapeutic efficacy. However, notable limitations and gaps in the research suggest areas for improvement and future exploration.

### 4.1. Summary of Postoperative and Neuropathic Pain Control Findings

The reviewed studies consistently showed that sodium-selective channel blockers can effectively reduce pain to levels comparable with or better than existing pain management options. For acute postoperative pain, VX-548 demonstrated superior efficacy to placebo and opioid controls, with fewer discontinuations due to lack of efficacy. Similarly, lacosamide, vixotrigine, ambroxol, and BIIB074 were effective for neuropathy and neuralgia, with varying levels of dose-dependent side effects such as dizziness, gastrointestinal symptoms, or mild local reactions.

The studies also highlight sodium-selective channel blockers’ potential for targeting specific pain mechanisms. For example, BIIB074, a Na_V_1.7-selective blocker, demonstrated a positive effect on trigeminal neuralgia, a condition characterized by heightened pain sensitivity. Ambroxol provided localized relief in CRPS patients via a topical formulation that minimizes systemic side effects. These findings collectively suggest that sodium-selective channel blockers can address distinct pain modalities, tailoring treatment to the underlying pain mechanisms.

### 4.2. Public Health Implications in the Context of the Opioid Epidemic

In the context of the ongoing opioid crisis, it is imperative to acknowledge the harmful consequences of widespread prescription of opioid analgesics for postoperative pain control. Overprescription carries unintended and dangerous consequences, such as increased risk of unintentional overdose among patients receiving opioid prescriptions [45]. In plastic and reconstructive surgery specifically, patients undergoing outpatient procedures such as rhinoplasty, breast surgery, and hand surgery are often left with a significant quantity of unused pills after recovery [46]. This overprescription not only exposes individual patients to the risk of misuse but also contributes to the broader societal problem of opioid diversion. Studies have found no significant differences in pain scores between patients who did or did not use their prescribed opioids after breast reduction and reconstruction surgeries, suggesting that many of these prescriptions are unnecessary [47].

Incorporating sodium-selective channel blockers into Enhanced Recovery After Surgery (ERAS) protocols can help reduce opioid prescriptions while ensuring effective pain management. Compared with traditional opioids, sodium-selective channel blockers have a safer side-effect profile, with mostly mild and transient adverse effects such as dizziness and gastrointestinal upset. They also lack addictive properties, as they do not activate reward pathways [9]. Recent advancements in opioid formulations, such as Naldebain (nalbuphine extended-release injection), offer an alternative with longer pain relief (up to seven days) and a lower risk of respiratory depression and addiction compared with conventional opioids. However, as a κ-opioid receptor agonist and a μ-opioid receptor antagonist, Naldebain still carries some opioid-related risks, whereas sodium-selective channel blockers eliminate opioid exposure entirely. Both approaches contribute to opioid-sparing pain management, and their use should be tailored to patient needs. While Naldebain extends pain relief with fewer opioid-related risks, sodium-selective channel blockers provide a completely opioid-free alternative, making them particularly valuable in high-risk populations [48].

### 4.3. Research Gaps and Areas for Improvement

While the reviewed studies demonstrate the promise of sodium-selective channel blockers, the current body of research is limited in scope and generalizability. Small sample sizes, particularly in studies like the ambroxol case series, reduce the reliability of findings, and short follow-up periods preclude an understanding of long-term safety and efficacy. Most studies lack head-to-head comparisons with commonly used analgesics, such as NSAIDs and acetaminophen, leaving their relative effectiveness unclear. Many studies revealed dose-dependent side effects, such as dizziness or gastrointestinal distress, that can impact patient adherence. Future research should focus on refining dosing strategies to maximize efficacy while minimizing adverse effects.

Notably, there remains a critical gap in the study of sodium-selective channel blockers specifically for postoperative pain. This is especially evident in our inclusion of only one article examining postoperative pain after only two types of procedures. The findings, while positive, were limited to bunionectomy and abdominoplasty, which have vastly different baseline levels of pain. Additional clinical trials may consider more common procedures, such as cholecystectomy and hernia surgery, to produce more generalizable results.

The included studies exclusively utilized a unimodal approach to pain management with sodium-selective channel blockers. The role of these drugs in multimodal pain regimens is not yet understood. Multimodal pain regimens incorporate multiple pharmacologic and non-pharmacologic therapies, like heat and massage, to minimize opioid exposure and optimize pain relief. Importantly, the mechanism by which acute pain evolves into chronic pain after surgery is unknown and likely constitutes more than one pathway. Unlike standard pain management, multimodal treatment accommodates this gap in knowledge by combining multiple approaches to cover more than one pain pathway. In theory, this decreases the chances that acute postoperative pain will transition to chronic pain [49]. Multimodal analgesia is also preferable to standard pain management because of fewer adverse effects, shorter length of hospital stay, and decreased opioid prescription [50]. Sodium-selective channel blockers are evidently effective when administered alone, but future studies need to prove their safety and utility alongside other pain relievers before they can be used in multimodal pain management. The cooperation of non-opioid analgesics, opioid analgesics, co-analgesics, and noninvasive treatments is multifaceted: dose titrations, routes of administration, schedules, and side effects are among the many elements to consider. We recommend future endeavors expand on the included articles to explore the interactions of topical and oral sodium-selective channel blockers with other analgesics. Data highlighting drug interactions and cumulative pain relief will be instrumental to safely and effectively incorporating sodium-selective channel blockers into multimodal pain interventions.

Research groups interested in multimodal pain management may take special interest in the potential of sodium-selective channel blockers to reduce opioid prescriptions. A 2017 analysis of opioid prescription dose and postoperative outcomes in orthopedic surgery found significant dose-dependent increases in postoperative complications for patients using opioids. Compared with the lowest dose quartile, the highest opioid dose quartile carried significantly higher odds of deep venous thrombosis, postoperative infection, and other complications, as well as increased cost and length of stay [51]. Opioid dosage and associated effects can be mitigated by multimodal analgesia; in fact, opioid use is inversely related to the number of non-opioid analgesics used [50]. Therefore, incorporating sodium-selective channel blockers as a new alternative in multimodal pain regimens may decrease opioid use and consequently improve patient well-being in the perioperative period. Interested groups may quantify how much sodium-selective channel blockers can reduce opioid prescription in multimodal analgesia while achieving comparable pain relief.

Sodium-selective channel blockers are an exciting new tool in the pain management landscape. Data strongly support their effectiveness in treating neuralgia, but their utility in postoperative pain management needs further examination. For both indications, present studies are deficient in scope and scale, warranting larger sample sizes and broader applications. Future studies may notably contribute by focusing on acute postoperative pain in more common surgeries, investigating the role of sodium-selective channel blockers in multimodal analgesia regimens, and exploring their potential to reduce opioid use in both inpatient and outpatient settings.

## 5. Conclusions

Sodium channel-selective analgesics represent a promising alternative or adjunct to opioid analgesics for postoperative pain as well as chronic neuropathic pain management. While current evidence supports their efficacy in reducing pain, particularly in neuropathic conditions, more extensive research is needed to confirm their effectiveness in acute postoperative settings. Incorporating sodium channel-selective analgesics into multimodal analgesia regimens may enhance pain control while mitigating the risks associated with opioid misuse and dependence. As the medical community continues to seek effective non-opioid analgesics, sodium channel blockers hold significant potential to improve patient outcomes and transform pain management practices.

## Figures and Tables

**Table 1:** Study characteristics.

Author, Year	Country	Study Type	Study Drug	Drug Target	N	% Women	Age (Mean ± SD)	Condition
de Greef, 2019 [40]	Netherlands	RCT	Lacosamide	Na_V_1.7	24	58.3%	54	Small fiber neuropathy
Maihöfner, 2018 [43]	Germany	Case Series	Ambroxol	Na_V_1.8	8	87.5%	47.25	Complex regional pain syndrome
Jones, 2023 [42]	USA	RCT	VX-548	Na_V_1.8	577	92.2%	46.01	Post-abdominoplasty or bunionectomy
Faber, 2023 [41]	UK	RCT	Vixotrigine	Na_V_1.7	122	45.9%	59.5	Small fiber neuropathy
Zakrzewska, 2017 [44]	UK	RCT	BIIB074	Na_V_1.7	29	65.5%	54.5	Trigeminal neuralgia

**Table 2:** Study outcomes.

Study	Study Drug	Controls	Key Metrics	Efficacy	Adverse Events	Primary Conclusion	NIH Quality Assessment
de Greef [40]	Lacosamide	Placebo	PI-NRS	58.3% of patients had ≥1-point reduction in pain vs. 21.7% placebo (*p* < 0.001)	Dizziness (37.5% 200 mg BID, 62.5% at 300 mg BID) Gastrointestinal symptoms (25% at 300 mg BID)	Effective for SFN with tolerable dose dependent side effects	Good
Maihöfner [43]	Ambroxol	None	PI-NRS	Average pain reduction of 3.0 points at rest and 3.5 during activity	No systemic adverse events, localized topical application was well tolerated	Promising topical treatment for early stage CRPS	Fair
Jones [42]	VX-548	Placebo, Hydrocodone-acetaminophen	NPRS	Significant pain reduction over 48 h with high-dose VX-548 (*p* < 0.05 vs. placebo)	Headache (6–8%) Constipation (3–4%)	Effective for acute postoperative pain with manageable side effects	Good
Faber [41]	Vixotrigine	Placebo	ADP, PGIC	Significant pain reduction with 200 mg BID (ADP: −0.85, *p* = 0.050); 350 mg BID not significant	Muscle spasms (7.3%) Falls (5.0%) Nasopharyngitis (6.7%)	Effective at moderate doses for diabetic SFN; higher doses limited	Good
Zakrzewska [44]	BIIB074	Placebo	CGIC, PI-NRS	45% placebo-adjusted reduction in paroxysms (*p* = 0.028); 50% placebo-adjusted reduction in ADP (*p* = 0.0009)	Headache (7%) Nasopharyngitis (7%) Sleep disturbances (7%)	Effective for trigeminal neuralgia; well tolerated	Fair
